# Isolated nerve palsy following insertion of a three-piece inflatable penile prosthesis

**DOI:** 10.1259/bjrcr.20210158

**Published:** 2021-12-17

**Authors:** Paulina Kosk, Alastair McKay, Arthur McPhee, David Cowell, Michael Fraser

**Affiliations:** 1 Clinical Development Fellow, Urology Department, Glasgow Royal Infirmary, Glasgow, UK; 2 Glasgow Royal Infirmary, Glasgow, UK; 3 Addenbrooke’s Hospital in Cambridge, Cambridge, UK

## Abstract

Case report of a 57-year-old male who underwent insertion of an inflatable penile prosthesis due to erectile dysfunction, secondary to poorly controlled Type 2 diabetes and Peyronie’s disease. The surgical procedure was uneventful and there were no immediate post-operative complications. During a routine follow-up, the patient described problems with the deflation of the implant and severe lower back and leg pain. Diagnostic MRI scans revealed reservoir migration, impingement of the obturator nerve and oedema in the adductor muscle group. The reservoir was initially repositioned, and later on removed due to ongoing symptoms.

## Clinical presentation

A 57-year-old male underwent insertion of an inflatable penile prosthesis (IPP) for erectile dysfunction (ED) secondary to poorly controlled Type 2 diabetes and Peyronie’s disease. A Coloplast Titan Touch^®^ with 18 cm cylinders was inserted via a penoscrotal incision along with a 125 ml reservoir (filled to 75 ml) in the right extraperitoneal space. There were no immediate post-operative complications and the patient was discharged home the following day.

The patient was seen for routine follow-up in the outpatient clinic after 6 weeks. The patient reported some difficulty deflating the device and was also complaining of persistent right-sided lower back, hip and leg pain. There was evidence of muscle wasting in the medial aspect of the thigh and power was Grade 4/5 (MRC Scale). An MRI of the penis and pelvis was performed.

## Imaging findings

**Figure 1.**
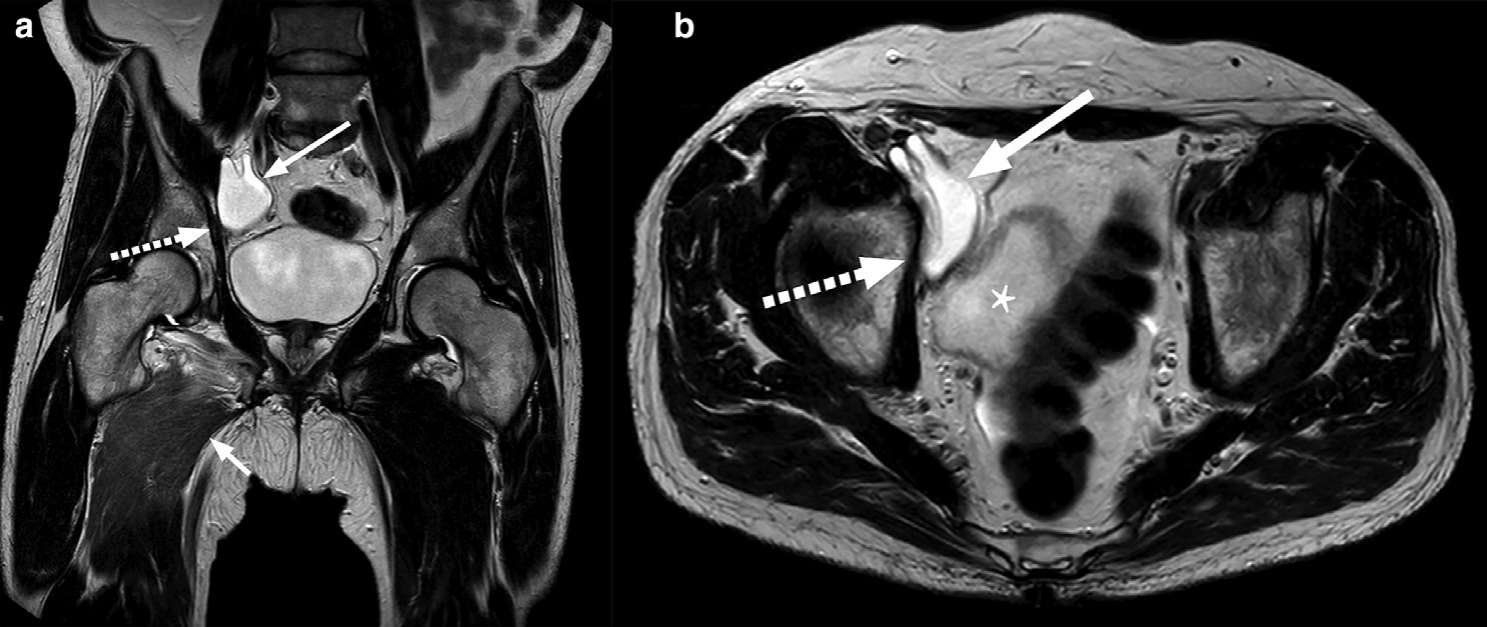
MRI T2 TSE in coronal (a) and transverse (b) views, demonstrate the intra-abdominal reservoir (arrow) compressed between the right pelvic side-wall (dashed arrow) and bladder (asterisk, b). This is the presumed site of the obturator nerve compression, immediately proximal to its entrance into the obturator foramen. We can also observe high signal oedema in the right adductor musculature (short arrow, a) in keeping with denervation oedema secondary to compression of the obturator nerve. TSE, turbo spin echo.

**Figure 2.**
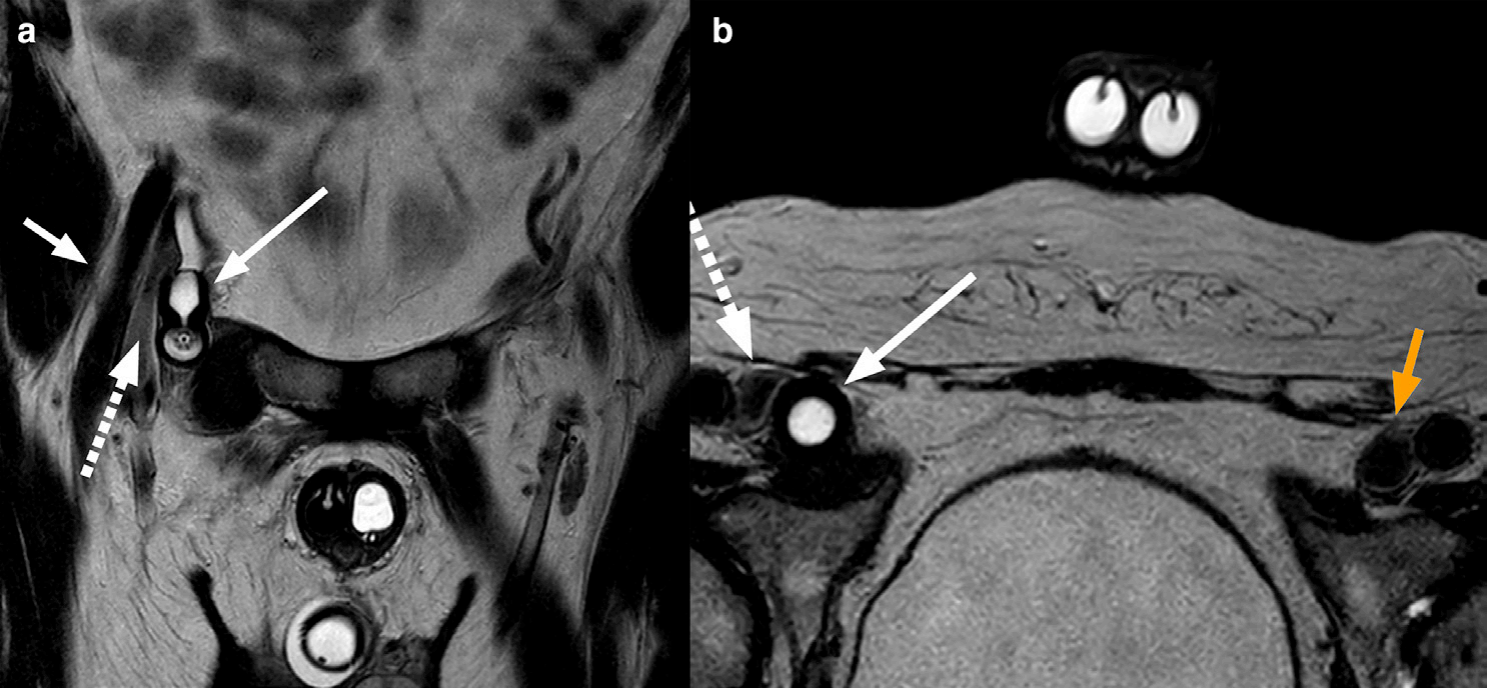
MRI T2 TSE in coronal (a) and transverse (b) views show the neck of the reservoir (arrow) extending into the femoral canal with compression of the right common femoral vein (dashed arrow), compared with the contralateral side (orange arrow, b). The femoral nerve lies laterally (short arrow, a) and is not compressed by the reservoir. A cross-section of the inflatable penile implant and scrotal pump are also visible. TSE, turbo spin echo.

**Figure 3.**
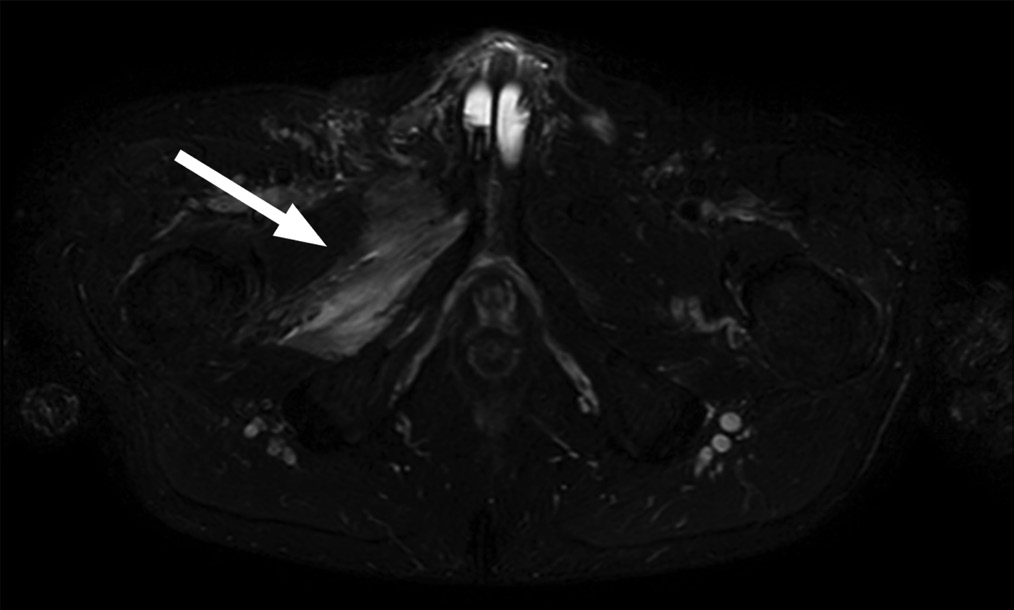
Transverse STIR sequence demonstrating high signal within the right adductor brevis and adductor magnus muscles (arrow) indicating denervation oedema. STIR, short-tau inversion recovery.

The MRI revealed that the reservoir had migrated from it’s initial position in Retzius space, into the right pelvic sidewall (Figure 1a and b) where it was causing an impingement of the right obturator nerve. Additionally, the neck of the reservoir was extending into the femoral canal and caused compression of the right common femoral vein (Figure 2a and b). There was associated oedema noted in the hip adductor muscle group, indicating denervation oedema secondary to the compression of the obturator nerve (Figures 1a and 3).

## Treatment

The patient went on to have the reservoir resited in an ectopic position. This procedure was later followed by a complete removal of the penile prosthesis as the patient continued to experience back and leg pain. Unfortunately, the patient has ongoing symptoms of nerve damage and suffers with life-long consequences including decreased mobility and persistent pain.

## Discussion

ED has a prevalence of approximately 10–20% and it is often an important early marker of systemic vascular disease. Insertion of a penile prosthesis is the surgical treatment of choice for medication resistant, irreversible ED. Patient satisfaction is high following surgery and complications are relatively rare.

There are two main types of IPP: two piece and three piece. The three-piece IPP has a separate reservoir which is traditionally placed in the extraperitoneal space (of Retzius) accessed via opening of the transversalis fascia at the superficial ring of the inguinal canal. The surgical maneuvers are performed to create a space for the reservoir in the extraperitoneal space and the reservoir is carefully placed within this space.

Blind placement of the reservoir remains a challenging step in the penoscrotal approach to three-piece IPP insertion. The most common complications after the IPP insertion include infection, mechanical failure, device erosion, corporal and urethral perforation, corporal cross-over and glans bowing. Migration of the reservoir is a rare complication with a reported rate of 0.7% , but can cause devastating consequences which might require surgical intervention.^1^ The most common areas of implant migration are the inguinal canal and subcutaneous plane but others have reported migration involving the scrotum, bladder, small and large bowel, and blood vessels.^2–4^ Migration often results in pain, poor refilling of the reservoir as it is compressed against a nearby structure, and the patient may complain of difficulty in device deflation. Additionally, certain patient factors such as prior robotic-assisted laparoscopic prostatectomy or radical cystectomy can further complicate the anatomy, by violating the space of Retzius, and increase the chance of post-operative complications. Lack of surgical experience is also correlated with the inappropriate positioning of the reservoir and a higher risk of post-surgical complications.^5^

The obturator nerve originates from L2, 3 and 4 lumbar spinal nerves before descending through the psoas muscle, over the pelvic brim and along the lateral pelvic wall. It passes through the obturator foramen in the pelvis and then divides into anterior and posterior branches. The anterior branch supplies the adductor brevis, longus and gracilis muscles and sensory innervation to the medial thigh. The posterior branch innervates the obturator externus and a portion of the adductor magnus, which is partially supplied by the sciatic nerve. Sensory branches also contribute to the knee joint sensation. The obturator nerve is located laterally and inferiorly to the space of Retzius and shouldn’t be compressed by a correctly placed IPP reservoir. Presented denervation oedema appeared in both adductor brevis and magnus muscles which suggests nerve compression within the pelvis, before it’s division into the branches.

Injury to the obturator nerve is rare and occurs mostly during gynaecological and urological procedures, which involve extensive retroperitoneal manipulation in obturator fossa.^6^ The most common presenting symptom of obturator neuropathy is that of altered sensation in the medial thigh – either that of paraesthesia or pain, and hip adduction weakness.^7^ As far as the authors are aware, this is the first reported case of IPP reservoir migration causing an isolated obturator nerve palsy.

MRI is the preferred imaging modality when it comes to the evaluation of a penile prosthesis. It has the ability to depict soft-tissues, penile anatomy and to provide detailed images of the prosthesis including the pelvic reservoir and scrotal pump. It also has the advantage of being free of ionising radiation and the ability to demonstrate anatomy on three orthogonal planes. To obtain the best quality of images, the patient should be in a supine position with penis placed, and stabilised with tape, in an anatomic position on anterior abdominal wall.^8^ *T*_2_ weighted images without fat suppression show the best definition between anatomical structures and IPP components, and are the most helpful sequence to diagnose implant complications.^9^ MRI is frequently used to confirm the presence of nerve entrapment or compression, to identify the cause of neuropathies. However, direct visualisation of the compressed structure is not always possible, like in this case. In this situation, good anatomic knowledge of the peripheral nerves innervation and indirect signs of obturator nerve impingement such as denervation edema in the adductor muscle group are sufficient to confirm the diagnosis.^10^

## Learning points

- Reservoir migration is a rare but important complication of three-piece IPP insertion with multiple potential sequelae.
- Injury to the obturator nerve is unusual and occurs mostly during gynaecological and urological procedures.
- The obturator nerve innervates the adductor muscle group. The adductor magnus and pectineus muscles have dual innervation.
- MRI provides an excellent adjunct to clinical examination in identifying reservoir migration where clinical suspicion exists. *T*_2_ weighted images are the best to review the IPP components.
- MRI images can be used to map out denervated muscles and thus localize and diagnose nerve entrapment.
- As far as the authors are aware, this is the first reported case of IPP reservoir migration causing an isolated obturator nerve palsy.
